# Effective connectivity changes in LSD-induced altered states of consciousness in humans

**DOI:** 10.1073/pnas.1815129116

**Published:** 2019-01-28

**Authors:** Katrin H. Preller, Adeel Razi, Peter Zeidman, Philipp Stämpfli, Karl J. Friston, Franz X. Vollenweider

**Affiliations:** ^a^Neuropsychopharmacology and Brain Imaging, Department of Psychiatry, Psychotherapy and Psychosomatics, University Hospital for Psychiatry Zurich, 8006 Zurich, Switzerland;; ^b^The Wellcome Centre for Human Neuroimaging, University College London, WC1N 3AR London, United Kingdom;; ^c^Monash Institute of Cognitive and Clinical Neurosciences, Monash University, Clayton, 3168 VIC, Australia;; ^d^Monash Biomedical Imaging, Monash University, Clayton, 3168 VIC, Australia;; ^e^Department of Electronic Engineering, NED University of Engineering and Technology, 75270 Karachi, Pakistan;; ^f^Department of Psychiatry, Psychotherapy and Psychosomatics, University Hospital for Psychiatry Zurich, 8006 Zurich, Switzerland;; ^g^Medical Research Center of the Department of Psychiatry, Psychotherapy and Psychosomatics, Psychiatric Hospital of the University of Zurich, 8006 Zurich, Switzerland;; ^h^Department of Child and Adolescent Psychiatry, Psychiatric Hospital of the University of Zurich, 8006 Zurich, Switzerland

**Keywords:** serotonin, LSD, fMRI, effective connectivity, spectral dynamic causal modeling

## Abstract

Psychedelics exert unique effects on human consciousness. The thalamic filter model suggests that core effects of psychedelics may result from gating deficits, based on a disintegration of information processing within cortico–striato–thalamo-cortical (CSTC) feedback loops. To test this hypothesis, we characterized changes in directed (effective) connectivity between selected CTSC regions after acute administration of lysergic acid diethylamide (LSD), and after pretreatment with Ketanserin (a selective serotonin 2A receptor antagonist) plus LSD in a double-blind, randomized, placebo-controlled, cross-over study in 25 healthy participants. We used spectral dynamic causal modeling (DCM) for resting-state fMRI data. Fully connected DCM models were specified for each treatment condition to investigate the connectivity between the following areas: thalamus, ventral striatum, posterior cingulate cortex, and temporal cortex. Our results confirm major predictions proposed in the CSTC model and provide evidence that LSD alters effective connectivity within CSTC pathways that have been implicated in the gating of sensory and sensorimotor information to the cortex. In particular, LSD increased effective connectivity from the thalamus to the posterior cingulate cortex in a way that depended on serotonin 2A receptor activation, and decreased effective connectivity from the ventral striatum to the thalamus independently of serotonin 2A receptor activation. Together, these results advance our mechanistic understanding of the action of psychedelics in health and disease. This is important for the development of new pharmacological therapeutics and also increases our understanding of the mechanisms underlying the potential clinical efficacy of psychedelics.

## Significance

Lysergic acid diethylamide (LSD) is a psychedelic drug that reliably induces an altered state of consciousness. Interest in psychedelic compounds is growing due to their remarkable potential for understanding altered neural states and potential clinical applications. However, there are major knowledge gaps regarding LSD’s neuropharmacology. Using cutting-edge neuroimaging methods we investigated directed connectivity between cortico–striato–thalamo-cortical (CSTC) regions after administration of LSD together with the specific role of the serotonin 2A receptor. Our results provide evidence that LSD alters directed connectivity within CSTC pathways in humans, suggesting that a disintegration of information processing within these loops is underlying the psychedelic state. These results inform the neurobiology of altered states of consciousness with critical implications for rational development of novel treatments.

Classic hallucinogens or psychedelics induce an altered state of consciousness characterized by alterations in mood, sensory perception, thought, and the sense of self (1). Psychedelics, therefore, offer the unique opportunity to investigate the neuropharmacological and mechanistic underpinnings of perception, thought, and consciousness. However, despite renewed scientific and clinical interest in these substances, there are still major gaps in our knowledge regarding the effects of psychedelics on the brain and their pharmacological mechanism of action (2).

Geyer and Vollenweider (3) proposed that key effects of psychedelics may result from gating deficits, based on the disintegration of information processing of internal and external stimuli within cortico–striato–thalamo-cortical (CSTC) feedback loops. This CSTC model suggests that the thalamus (Thal) plays a key role in controlling or gating information to the cortex and is thereby critically involved in the regulation of consciousness (3). Alterations beyond the normal range of thalamic gating of information are suggested to result in an overload of the cortex, with excessive exteroceptive and interoceptive stimuli that may ultimately cause the sensory flooding, cognitive disruptions, and ego dissolution seen in both naturally occurring psychoses and drug-induced altered states of consciousness (3, 4). This thalamic-gating model is supported by results obtained in schizophrenia patients showing increased functional and effective connectivity between the thalamus and specific cortical brain regions (5, 6) and studies showing deficits in preattentive sensorimotor gating after the administration of psychedelics (7–9).

Thalamic gating has been suggested to be influenced by several neurotransmitter systems. Blockade of NMDA receptors, increase of dopaminergic neurotransmission, or excessive stimulation of serotonin (5-HT) 2A receptors could lead to a neurotransmitter imbalance within CSTC loops, which results in an opening of the thalamic filter. The mesostriatal dopaminergic and serotonergic projections provide input to the striatum and are thought to be counterbalanced by the glutamatergic input derived from cortico–striatal pathways. Alterations in these neurotransmitter systems may result in a diminished influence of the striatum on the thalamus and open the thalamic filter (3, 4).

However, so far this model and its underlying pharmacology have never been tested in humans. Pharmacological neuroimaging offers the opportunity to address these knowledge gaps by investigating the influence of psychedelics on CSTC loops and elucidating specific receptor contributions. Lysergic acid diethylamide (LSD) is a prototypical psychedelic drug with predominantly agonist activity at 5-HT-2A/C, -1A/B, -6, and -7, and dopamine D2 and D1 receptors (10). In this study, administration of LSD with and without pretreatment with the selective 5-HT_2A_ receptor antagonist Ketanserin (Ket) offered the unique opportunity to investigate the role of 5-HT_2A_ receptor stimulation by LSD in potentially altering the integration within and between key constituents of CTSC system: the thalamus, the ventral striatum (VS), the posterior cingulate cortex (PCC), and the superior temporal gyrus. The PCC and the temporal cortex (Temp) are two cortical areas that have repeatedly been identified to play a major role in psychedelic-induced altered states of consciousness, in particular in alterations of self-experience (11–14).

**Fig. 1.**
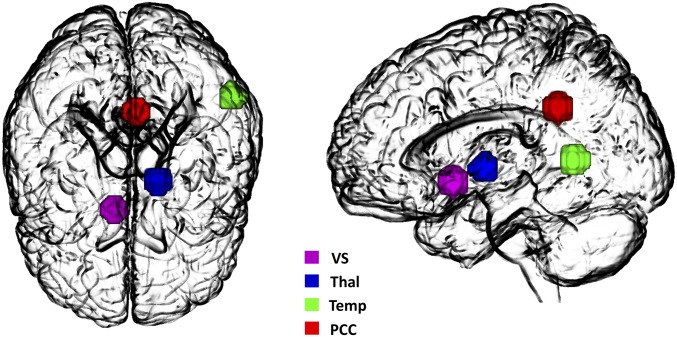
Four nodes of the DCM. The model includes the following nodes: VS, Thal, Temp, and PCC. The associated time series were used to invert the spectral DCM with a fully connected architecture.

Furthermore, the present study capitalizes on recent advances in modeling the endogenous brain activity, which underlies resting-state fMRI data. We characterized each subject’s distributed neuronal dynamics using spectral dynamic causal modeling (DCM) (15), which rests on a generative model of how interacting neural populations cause fMRI time series, which in turn give rise to functional connectivity measures (i.e., correlated hemodynamic fluctuations) (Fig. 1). DCM provides estimates of parameters quantifying the strength of directed connections between regions. We estimated these parameters at the between-condition level using a Bayesian model (parametric empirical Bayes, PEB) (16), enabling us to test for between-session (i.e., drug) effects. Compared with functional connectivity approaches, which provide measures of correlation that cannot be used to infer causality, spectral DCM quantifies (directed) effective connectivity among brain regions. This aspect is crucial for testing the thalamic-gating model, which predicts LSD-induced increase in connectivity from the thalamus to cortical regions and decreased connectivity from the VS to the thalamus.

This study, therefore, constitutes an investigation of fMRI effective resting-state connectivity within the CTSC loops, its alterations by LSD, and the underlying receptor pharmacology. We hypothesized LSD-induced changes of effective connectivity in accordance with the predictions of the thalamic-gating model. Furthermore, we previously reported that psychedelic effects in humans could be blocked by 5-HT2A receptor antagonism (17, 18). Therefore, we hypothesized that LSD-induced connectivity changes are predominantly dependent on the 5-HT2A receptor.

## Results

### Subjective Drug Effects.

Subjective drug effects were assessed using the 5-Dimensions Altered States of Consciousness (5D-ASC) questionnaire (19). Detailed results are reported elsewhere (17). In brief, all LSD-induced subjective drug effects were blocked by Ket. No significant differences were found between the placebo (Pla) and the Ket + LSD conditions (*SI Appendix*, Fig. S1).

### Drugs vs. Placebo.

**Fig. 2.**
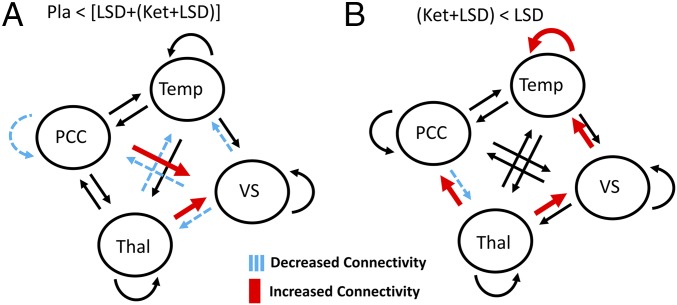
Connectivity changes for (*A*) Pla < [LSD + (LSD + Ket)] and (*B*) (LSD + Ket) < LSD. *n* = 25.

The contrast drug vs. placebo [Pla < (LSD + [Ket + LSD])] resulted in increased effective connectivity from the thalamus to the VS and the PCC to the VS. Furthermore, decreases in effective connectivity were found: from the thalamus to the Temp, the VS to thalamus, the VS to PCC, and the VS to Temp. Regarding inhibitory self-connections, which control the regions’ gain or sensitivity to inputs, there was reduced self-inhibition (i.e., disinhibition) of PCC due to the drug. These results are shown in Fig. 2*A*.

### 5-HT2A Blockade.

The contrast Ket + LSD < LSD revealed increased effective connectivity from the thalamus to the VS and PCC, and from the VS to the Temp. Decreased effective connectivity was found from the PCC to the thalamus. Furthermore, we found an increase in self-inhibition of the Temp. These results are shown in Fig. 2*B*.

An additional advantage of DCM is the possibility to determine the valence of the connections: that is, define whether they are excitatory or inhibitory in nature. See Table 1 for the summary of the signed connectivity parameters and *SI Appendix*, Table S1 for parameter estimates (mean and variance).

**Table 1.** Summary of results

Connection	Connectivity: ↑ = increased, ↓ = decreased	Valence: + = excitatory, − = inhibitory	Effect size (typical effect size is 0.1 Hz)
Contrast 1: Placebo < [LSD + (Ket + LSD)]			
Thal → VS	↑	+	0.088
PCC → VS	↑	+	0.047
Thal → Temp	↓	+	0.275
VS → Thal	↓	+	0.184
VS → PCC	↓	+	0.139
VS → Temp	↓	−	0.325
PCC → PCC	↓	−	0.091
Contrast 2: (Ket + LSD) < LSD			
Thal → VS	↑	+	0.143
Thal → PCC	↑	+	0.276
VS → Temp	↑	−	0.169
PCC → Thal	↓	−	0.098
Temp → Temp	↑	−	0.18

*n* = 25.

In sum, our analyses showed that LSD increased effective connectivity from the thalamus to the PCC, whereas the reciprocal connection from the PCC to the thalamus had reduced effective connectivity. Furthermore, we also found decreased excitability (i.e., increased self-inhibition) in the Temp. These alterations seem to be driven by 5-HT_2A_ receptor stimulation by LSD because they were blocked by Ket. Furthermore, we found that LSD decreased effective connectivity from the VS to the thalamus and the PCC, from the thalamus to the Temp, increased effective connectivity from the PCC to the VS, and reduced self-inhibition of the PCC. These LSD-induced changes were not blocked by Ket.

## Discussion

Psychedelics induce an altered state of consciousness that, on the one hand, mimics predominantly positive symptoms of schizophrenia, but on the other hand, may have beneficial therapeutic effects in mood and anxiety disorders (20). The history of research on psychedelics is also intertwined with the investigation of the role of the serotonergic system in cognition and behavior in health and psychiatric disorders (3). However, the neurobiology and pharmacology of psychedelics, in particular LSD, is still poorly understood in humans. Geyer and Vollenweider (3) proposed that key effects of psychedelics may result from the disintegration of information processing of internal and external stimuli within CSTC feedback loops. In this work we sought to mechanistically test this hypothesis by leveraging the recent advances in modeling endogenous activity using DCM for resting-state fMRI (15) with the administration of LSD and Ket, a 5-HT_2A_ receptor antagonist. Investigating effective (resting-state) connectivity in LSD-induced states, the present study closes critical knowledge gaps by showing that LSD alters connectivity within CSTC pathways. In particular, LSD increases effective connectivity from the thalamus to cortical areas, via agonistic activity on the 5-HT_2A_ receptor, and decreases effective connectivity from the VS to the thalamus independently of 5-HT_2A_ receptor stimulation.

At the core of the CSTC model by Geyer and Vollenweider (3) is the hypothesis that psychedelics alter the capacity of the thalamus to control or gate the flow of information to the cortex. The thalamus is the central part of the diencephalon containing relay cells that project to the cortex (21). The thalamus also gates the main input to the cortex from subcortical areas and likely all regions of the cortex receive input from the thalamus (21). It also plays a key role in various neurobiological theories of consciousness, suggesting that neural activity in thalamo-cortical loops gives rise to conscious experience (22, 23). During sleep, sedation, and anesthesia, thalamo-cortical connectivity is decreased, while information transfer from the thalamus to the cortex is highest during states requiring high levels of sustained attention (24–29). Alterations in thalamo-cortical connectivity are also crucial features of various psychiatric disorders, predominantly schizophrenia (6, 30–34), and also depression and obsessive-compulsive disorder (35).

Here, we show that, in line with the CSTC model, LSD increased the excitatory connection from the thalamus to the PCC. This finding is particularly interesting in light of predictive coding formulations of the CSTC model, where filtering or gating is implemented via changes in the precision of ascending prediction errors. Much neurobiological evidence points to the thalamus as a key source of the requisite neuromodulation (36). This result is also consistent with a previous study showing increased functional thalamo-cortical connectivity after LSD administration (37). Furthermore, LSD increased excitability of the PCC and reduced effective connectivity from the PCC to the thalamus. The PCC has repeatedly been shown to be involved in the effects of psychedelics. For example, decreased functional connectivity between the PCC and frontal brain areas has been reported after psilocybin and LSD administration (12, 14), as well as decreased global brain connectivity after LSD administration (38). Furthermore, at a postacute assessment 24 h after ayahuasca intake, functional connectivity was increased between the PCC and the anterior cingulate cortex and reductions in glutamate + glutamine, creatine, and *N*-acetylaspartate + *N*-acetylaspartylglutamate in the PCC were measured (39). Our study showing increased excitability of the PCC is also in line with previous results reporting LSD, psilocybin, and ayahuasca-induced decreases in α-power (13, 40–42), as decreases in α-oscillations have been reported to reflect a state of enhanced cortical excitability (43). Specifically, reductions in α-power in the PCC assessed with magnetoencephalography after psilocybin and LSD administration correlated with self-report questionnaire items indicating psilocybin-induced alterations in self-processing and ego-integration (13). This is in line with current theories of the role of the PCC in cognitive functioning, which associate the PCC with arousal and awareness as well as the control of the balance between internally and externally directed thought (44). The interaction of the PCC with other brain networks is therefore considered to be important for conscious awareness and the failure to suppress PCC activity to be associated with the intrusion of internal mentation (44). Previous effective connectivity studies using the spectral DCM have consistently shown the PCC, which is a core region in the default mode network (DMN), to be a robustly driven hub receiving information from other core regions of the DMN: that is, the medial prefrontal cortex and bilateral angular gyrus (45–47).

The CSTC model also proposes that the gating capacities of the thalamus are controlled by the striatum (4). Vollenweider and Geyer hypothesized that increasing serotonergic activation by psychedelics reduces the influence of the striatum on the thalamus, which leads to opening the thalamic filter. The present data show that LSD indeed reduces effective connectivity from the VS to the thalamus, therefore corroborating the assumption that alterations in striatal–thalamic interaction are important mechanisms underlying psychedelic states.

In contrast to LSD-induced increases in effective connectivity from the thalamus to the PCC, excitatory connectivity from the thalamus to the Temp was decreased and the inhibitory self-connection of the Temp was increased by LSD. Previous studies showed that: (*i*) decreases in functional connectivity between the Temp and other cortical regions after LSD and psilocybin administration (11, 12), and (*ii*) alterations in lagged phase synchronization of δ-oscillations after psilocybin administration (42), both correlating with subjective drug-induced effects. The temporal lobe is associated with processing emotional and social information (48), both of which are altered in psychedelic states (49–51) and is implicated in the pathophysiology of schizophrenia (52). Because it has been reported that patients suffering from major depression disorder show increased thalamo-temporal connectivity (53), attenuating effective connectivity from the thalamus to the Temp might additionally represent a neurobiological mechanism by which psychedelics potentially exert their antidepressive potential (54). However, because the present study investigated acute alterations in connectivity, further studies are needed to clarify whether these results translate into long-term therapeutic effects.

The present results therefore show that while the thalamus indeed decreases information gating and therefore increases “bottom-up” information flow to certain cortical areas as well as the VS in accordance with the CSTC model, LSD does not cause an undifferentiated cortical inundation as first hypothesized in the model, but rather leads to a pattern of increased information flow to particular areas of the cortex while thalamic connectivity with other cortical areas is reduced in resting state. This might explain the seemingly paradoxical subjective effects often reported in psychedelic-induced altered states of consciousness that are characterized by increased arousal as well as a dreamlike experience, impaired cognition but at the same time reported perceived mental clarity, and psychosis-like effects combined with blissful experiences (55). Therefore, psychedelics states differ from previously investigated states like anesthesia, sleep, or cognitively demanding situations (24–28).

The design of the present study also involved the pretreatment of LSD with the 5-HT_2A_ receptor antagonist Ket and therefore allowed for investigation of the role of this specific receptor system in LSD-induced alterations in effective connectivity. While we have previously shown that Ket blocked all subjective and most neural effects of LSD (17, 38), the present analysis showed particular alterations in the thalamus–PCC connections, dependent on the 5-HT_2A_ receptor. This is in line with a previous study showing that PCC desynchronization under the influence of psilocybin can be explained by increased excitability of 5-HT2A receptor-rich deep-layer pyramidal neurons (13). In our study, Ket blocked increased effective connectivity from the thalamus to the PCC and reduced connectivity from the PCC to the thalamus, as well as increased inhibition of the Temp. On the other hand, mostly connections involving the VS were not blocked by Ket: decreased effective connectivity from the VS to the thalamus and the PCC, from the thalamus to the Temp, increased effective connectivity from the PCC to the VS, and reduced inhibition of the PCC, suggesting that these LSD-induced alterations are probably not attributable to LSD’s agonistic action on the 5-HT_2A_ receptor. Previous animal studies suggested that, in addition to, 5-HT2A receptors, dopamine D2 receptors play a role in the effects of LSD (56, 57). Considering that the effects not blocked by Ket mostly involve the VS and that the striatum is a key structure in dopaminergic pathways, the involvement of the dopamine system in LSD-induced alterations in brain connectivity seems likely. However, the present study cannot conclusively answer questions regarding the role of other receptor systems beyond the 5-HT_2A_ system in the neurobiological effects of LSD, and therefore further studies are warranted to test the specific contributions of other receptors, for example, by pretreatment with dopamine-antagonists. However, considering that Ket normalized all subjective effects, it is, on the one hand, possible that alterations induced by LSD’s action on other receptors only provoke noticeable subjective effects when 5-HT_2A_ receptors are stimulated concurrently. On the other hand, it is possible that higher doses of LSD are needed to produce noticeable subjective effects even when the 5-HT_2A_ receptor is blocked. Future studies could therefore test the effects of multiple (and higher) doses of LSD.

Beyond the CSTC model, several authors have proposed additional theories to explain the biological underpinnings of alterations in consciousness experienced following the administration of psychedelics. An early psychophysiological model by Fischer proposed that shifts in the ratio of sensory-to-motor activity could explain subjective symptoms (58). More recent evidence from human studies suggests that psychedelic-induced alterations in consciousness are related to increased entropy in the brain, leading to disorganization of brain activity and more flexible cognition (entropic brain hypothesis) (59). This model has been corroborated with studies showing that psilocybin produces an increase in blood-oxygen level-dependent (BOLD) signal variance and decreased connectivity within the DMN (60, 61). These models are not mutually exclusive, and focus on complementary correlates of altered states of consciousness. While this is beyond the scope of the present study, a unifying framework should be developed and tested in future studies.

This study was limited to brain regions implicated by the CSTC model, which have been shown empirically to be sensitive to the effects of psychedelics in previous studies, as well as within the current participants (3, 4, 11–14, 17, 62). Clearly, testing additional models in future studies has the potential to extend our knowledge about the effects of LSD on effective connectivity among more distributed brain regions, in particular, additional hubs of the CSTC model such as the medial prefrontal cortex. Furthermore, future analyses using smaller regions-of-interest (ROIs) could reveal differential effects of subregions of the current ROIs. Additionally, to focus on drug effects using efficient estimates of the cross-spectral density (CSD), we concatenated time-series data over subjects. This precluded analysis between subject effects; for example, we were unable to relate connectivity parameters to subjective effects. We will address this limitation in the future using (hierarchical) PEB. Although a previous study has shown that Ket does not induce an altered state of consciousness or affect sensorimotor gating (8), the lack of a Ket + Pla condition represents a further limitation of this study. This suggests that future studies will be needed to characterize the influence of Ket alone on effective connectivity.

In sum, the present results confirm major predictions proposed in the CSTC model and provide evidence that LSD alters effective connectivity within CSTC pathways that have been implicated in the gating of sensory and sensorimotor information to the cortex: LSD diminishes the influence of the striatum on the thalamus and opens the thalamic filter, but selectively: only to certain areas of the cortex. In particular, the present results pinpoint the role of the thalamus–PCC connection for the effects of psychedelics. Additionally, the present results enhance our knowledge about the contribution of the serotonin system to the functional organization of the brain in LSD-induced states. Finally, the present study showcases the newly developed spectral DCM approach—coupled with modeling using the PEB framework—to testing hypotheses in resting-state pharmacological fMRI at the group level. Taken together, the results deepen our knowledge about the mechanism of action of psychedelics relevant for health and disease and important for the development of new pharmacological therapeutics.

## Methods

### Participants.

The data analyzed in this paper were collected as part of a larger study, which is reported in refs. 17 and 38. The present characterization of these data extends the analysis presented in Preller et al. (38). Participants were recruited through advertisements placed in local universities. All participants underwent a screening visit before inclusion in the study. All included participants were healthy according to medical history, physical examination, blood analysis, and electrocardiography. The Mini-International Neuropsychiatric Interview (MINI-SCID) (63), the *Diagnostic and Statistical Manual of Mental Disorders*, fourth edition self-rating questionnaire for Axis-II personality disorders (SCID-II) (64), and the Hopkins Symptom Checklist (SCL-90-R) (65) were used to exclude subjects with present or previous psychiatric disorders or a history of major psychiatric disorders in first-degree relatives. Participants were asked to abstain from the use of any prescription or illicit drugs for a minimum of 2 wk before the first test day and for the duration of the entire study, and to abstain from drinking alcohol for at least 24 h before test days. Urine tests and self-report questionnaires were used to verify the absence of drug and alcohol use. Participants were required to abstain from smoking for at least 60 min before MRI assessment and from drinking caffeine during the test day. Urine tests were also used to exclude pregnancy. Further exclusion criteria included left-handedness, poor knowledge of the German language, cardiovascular disease, history of head injury or neurological disorder, history of alcohol or illicit drug dependence, MRI exclusion criteria, including claustrophobia, and previous significant adverse reactions to a hallucinogenic drug.

Twenty-five participants took part in the study (*n* = 19 males and 6 females; mean age = 25.24 y; SD = 3.72 y; range = 20–34 y). All participants provided written informed consent statements in accordance with the declaration of Helsinki before participation in the study. Subjects received written and oral descriptions of the study procedures, as well as details regarding the effects and possible risks of LSD and Ket treatment. The Swiss Federal Office of Public Health, Bern, Switzerland, authorized the use of LSD in humans, and the study was approved by the Cantonal Ethics Committee of Zurich. The study was registered at ClinicalTrials.gov (NCT02451072).

### Study Design.

In a double-blind, randomized, cross-over design, subjects received either: (*i*) Pla + Pla condition: Pla (179 mg Mannitol and Aerosil 1 mg orally) after pretreatment with Pla (179 mg Mannitol and Aerosil 1 mg orally); (*ii*) Pla + LSD condition: LSD (100 µg orally) after pretreatment with Pla (179 mg Mannitol and Aerosil 1 mg orally); or (*iii*) Ket + LSD (Ket + LSD) condition: LSD (100 µg po) after pretreatment with the 5-HT2A antagonist Ket (40 mg orally) at three different occasions 2 wk apart. Pretreatment with Pla or Ket occurred 60 min before treatment with Pla or LSD to allow for Ket to reach peak plasma levels (66). The resting-state scans (10 min) were acquired twice: 75 min (session 1) and 300 min (session 2) after treatment administration. The 5D-ASC (a retrospective self-report questionnaire) (19) was administered to participants 720 min after drug treatment to assess subjective experience after drug intake. Ninety-four items are answered on visual analog scales. Scores were calculated for 11 validated scales (67): experience of unity, spiritual experience, blissful state, insightfulness, disembodiment, impaired control and cognition, anxiety, complex imagery, elementary imagery, audiovisual synesthesia, and changed meaning of percepts. The 5D-ASC score was analyzed using a repeated-measures ANOVA with drug condition (Pla, LSD, and Ket + LSD) and scale as within-subject factors.

### MRI Data Acquisition and Preprocessing.

MRI data were acquired on a Philips Achieva 3.0T whole-body scanner. A 32-channel receive head coil and MultiTransmit parallel radio frequency transmission was used. Images were acquired using a whole-brain gradient-echo planar imaging (EPI) sequence (repetition time, 2,500 ms; echo time, 27 ms; slice thickness, 3 mm; 45 axial slices; no slice gap; field of view, 240 × 240 mm^2^; in-plane resolution, 3 × 3 mm; sensitivity-encoding reduction factor, 2.0). Additionally, high-resolution anatomical images (voxel size, 0.7 × 0.7 × 0.7 mm) were acquired using a standard T1-weighted 3D magnetization prepared rapid-acquisition gradient echo sequence. The acquired images were analyzed using SPM12 (https://www.fil.ion.ucl.ac.uk). The preprocessing steps of the images consisted of slice time correction, realignment, spatial normalization to the standard EPI template of the Montreal Neurological Institute (MNI), and spatial smoothing using a Gaussian kernel of 6-mm full-width half-maximum. We investigated for any excessive head motion but head movement did not exceed 3 mm in any participant.

### ROI Selection and Time-Series Extraction.

This study aims at investigating the effect of LSD on the integration within and between key constituents of CTSC system. For this purpose, ROIs were identified as key nodes for effective connectivity analysis, based on previous literature that considered: (*i*) the thalamic gating model (3, 4); (*ii*) psychedelic-induced modulations of brain activity and functional connectivity (11–14, 62) in independent participant cohorts; and (*iii*) LSD-induced alterations in BOLD signal in the current cohort of participants, using (independent) task-based data (17, 18). The CTSC loop was comprised of the thalamus, the VS, the PCC, and the temporal gyrus. ROIs were masked by an 8-mm radius sphere centered on previously reported MNI coordinates of these regions. These MIN coordinates were derived from LSD-induced alterations in BOLD signal, in the same cohort of participants, using task-based data (17, 18): thalamus: *x* = −15, *y* = −8, *z* = 1; VS: *x* = 9, *y* = 8, *z* = −8; PCC: *x* = −3, *y* = −46, *z* = 31; Temp: *x* = −56, *y* = −54, *z* = 8. These ROIs are shown in Fig. 1. Time series from the four ROIs were corrected for head motion and physiological noise. For this purpose, the nuisance regressors included the six head-motion parameters, cerebrospinal fluid (extracted from left ventricle using a 4-mm sphere), and white matter (extracted from pons using a 4-mm sphere) regressors. Low-frequency signal drifts were filtered using a 128-s high-pass filter.

### Dynamic Causal Modeling.

The spectral DCM analyses were conducted using DCM12 (revision 6759) implemented in the SPM12 (revision 12.2). In brief, we concatenated the time series over subjects for each region, drug condition, and session. This enabled us to focus on the difference between drug conditions using more efficient estimates of the CSD. However, this precluded analysis of between-subject effects, such as subjective effects. These estimates were then taken to the between-condition level and modeled with two orthogonal contrasts of condition-specific effects; namely, Pla vs. drug conditions with and without Ket. This was repeated for both (early and late) sessions. In detail, for each drug condition and session, a fully connected model was created to compare all possible nested models of between- and within-region interactions (16). DCM was estimated using spectral DCM, which fits the complex cross-spectral density using a (parameterized) power-law model of endogenous neuronal fluctuations (15, 47). For more details, see the *SI Appendix*. This analysis provides measures of causal interactions between regions, as well as the amplitude of endogenous neuronal fluctuations within each region (47). Because the main effect of drug did not differ between sessions, we focus on the first (early) session. To investigate the (*i*) effect of drug and (*ii*) influence of the 5-HT_2A_ blockade by Ket, the following two orthogonal contrasts of effects (at the between-subject level) were modeled: Pla < [LSD + (Ket + LSD)] and (Ket + LSD) < LSD. To report important effects, we used model comparisons (using free energy) with and without each effect and then calculated the posterior probability for each model, which is simply a softmax function of the log Bayes factor. We only report effects (i.e., changes in directed connectivity) that have a posterior probability >0.95 (*SI Appendix*, Table S1).

### Data Availability.

The extracted time series for each ROI and each condition, as well as all statistics applied to these data, have been deposited in Bitbucket (68).

## Supplementary Material

Supplementary File
