# Supplementary material for: Effective connectivity changes in LSD-induced altered states of consciousness in humans
Source: Proc Natl Acad Sci U S A. 2019 Jan 28;116(7):2743–8. doi: 10.1073/pnas.1815129116 (PMC6377471; doi:10.1073/pnas.1815129116)
Supplement: Supplementary File [file pnas.1815129116.sapp.pdf]

PNAS
www.pnas.org

Supplementary Information for

Effective connectivity changes in LSD-induced altered states of consciousness in humans

Katrin H. Preller, Adeel Razib, Peter Zeidman, Philipp Stämpflie, Karl J. Friston, Franz X. Vollenweider

Corresponding Author: Katrin H. Preller
Email: preller@bli.uzh.ch

This PDF file includes:

Supplementary Methods
Fig. S1
Table S1

www.pnas.org/cgi/doi/10.1073/pnas.1815129116

Supplementary Methods:

Spectral Dynamic Causal Modelling

Dynamic causal modelling (DCM) is Bayesian framework that infers the directed (causal) connectivity among the neuronal systems – referred to as effective connectivity. A new DCM for resting state fMRI was recently proposed based upon a deterministic model that generates predicted cross spectra, referred to as spectral DCM. In order to model resting state activity – in the absence of external stimuli – a stochastic component capturing neural fluctuations is included in the model. Mathematically, we can express the formulation of the stochastic generative model as a set of two equations. First is the neuronal state equation, namely

$$\dot{x}(t) = f(x(t), u(t), \theta) + v(t), \qquad \text{(S1)}$$

and second is the observation equation, which is a static nonlinear mapping from the hidden physiological states in (1) to the observed BOLD activity and is written as:

$$y(t) = h(x(t), \varphi) + e(t), \qquad \text{(S2)}$$

where $\dot{x}(\mathrm{t})$ is the rate of change of the neuronal states $x(t)$, θ are unknown parameters (i.e. the effective connectivity) and $v(t)$ (resp. $e(t)$) is the stochastic process – called the state noise (resp. the measurement or observation noise) – modelling the random neuronal fluctuations that drive the resting state activity. In the observation equations, φ are the unknown parameters of the (haemodynamic) observation function and $u(t)$ represents any exogenous (or experimental) inputs that drive the hidden states – that are usually absent in resting state designs (1). Spectral DCM furnishes a constrained inversion of the stochastic model by parameterising the neuronal fluctuations $v(t)$. Spectral DCM simplifies the generative model by replacing the original timeseries with their second-order statistics (i.e., cross spectra). This means, instead of estimating time varying hidden states, we are estimating their covariance, which is time invariant. Then we simply need to estimate the covariance of the random

fluctuations; where a scale free (power law) form for the state noise (resp. observation noise) is used – motivated from previous work on neuronal activity (2-4) – as follows:

$$g_v(\omega,\theta) = \alpha_v \omega^{-\beta_v}$$

$$g_e(\omega,\theta) = \alpha_e \omega^{-\beta_e} \qquad \text{(S3)}$$

Here, $\{\alpha,\beta\} \subset \theta$ are the parameters controlling the amplitudes and exponents of the spectral density of the neural fluctuations. The parameterisation of endogenous fluctuations means that the states are no longer probabilistic; hence the inversion scheme is significantly simpler, requiring estimation of only the parameters (and hyperparameters) of the model.

We used standard Bayesian model inversion to infer the parameters of the model in (1), (2) and (3), from the observed signal $y(t)$. The description of the Bayesian model inversion procedures based on variational Laplace can be found elsewhere for the interested readers (5-7).

Parametric Empirical Bayes

Empirical Bayes refers to the Bayesian inversion or fitting of hierarchical models. In hierarchical models, constraints on the posterior density over model parameters at any given level are provided by the level above. These constraints are called empirical priors because they are informed by empirical data. A hierarchical Parametric Empirical Bayes (PEB) model for DCM parameters was recently introduced, which represents how individual (within-subject) connections derive from the subjects' group membership (8). Mathematically, for DCM studies with *N* subjects and *M* parameters per DCM, we have a hierarchical model, where the responses of the *i*-th subject and the distribution of the parameters over subjects can be modeled as:

$$y_i = \Gamma_i^{(1)}(\theta^{(1)}) + \varepsilon_i^{(1)} \qquad \text{(S4)}$$

$$\theta^{(1)} = \Gamma^{(2)}\left(\theta^{(2)}\right) + \varepsilon^{(2)}$$

$$\theta^{(2)} = \eta + \varepsilon^{(3)}$$

where, y_i is the BOLD time series from *i-th* subject *and* $\Gamma_i^{(1)}$ is a nonlinear mapping from the parameters of a model to the predicted response y, which in this study was the model in Eq. S1 above. $\varepsilon_i^{(1)}$ is independent and identically distributed (i.i.d.) observation noise (equivalent to $e(t)$ in Eq. S2). In this hierarchical form, *empirical priors* encoding second (between-subject) level effects place constraints on subject-specific parameters. The second level would be a linear model where the random effects are parameterised in terms of their precision:

$$\Gamma^{(2)}\left(\theta^{(2)}\right) = (X \otimes W)\beta$$

where, $\beta \subset \theta$ are group means or effects encoded by a design matrix with between X and within-subject W parts. The between-subject part encodes differences among subjects or covariates such as age, while the within-subject part specifies mixtures of parameters that show random effects. We assume that the first column of the design matrix is a constant term, modelling group means and subsequent columns encode group differences.

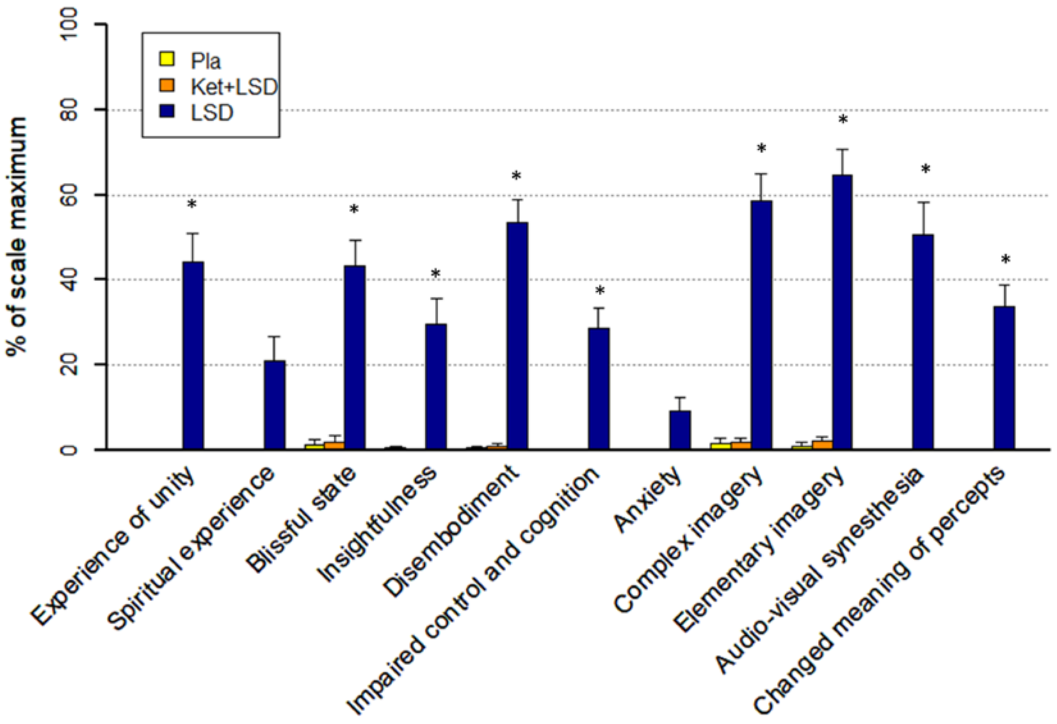

Fig. S1: Subjective drug effects. Retrospectively assessed 5D-ASC scores in the Placebo (Pla), Ketanserin+LSD (Ket+LSD), and LSD treatment conditions. Scores are expressed as a percent of the scale maximum. Scores in the LSD treatment condition differed significantly from Placebo and Ketanserin+LSD treatment conditions on each scale except for spiritual experience and anxiety (*$p < 0.05$, Bonferroni corrected; n = 25 participants).

Table S1. Summary of parameter values

Condition 1: Placebo < [LSD + (Ket+ LSD)]			
**Connection**	**Mean**	**Variance**	**Posterior probability**
Thal →VS	0.088	0.0005	1.0
PCC → VS	0.047	0.0001	1.0
Thal → Temp	0.275	0.0006	1.0
VS → Thal	0.184	0.0008	1.0
VS → PCC	0.139	0.0022	0.99
VS → Temp	0.325	0.0018	1.0
PCC → PCC	0.091	0.0008	0.99
**Condition 2: (Ket+LSD) < LSD**			
Thal → VS	0.143	0.0009	1.0
Thal → PCC	0.276	0.0036	1.0
VS → Temp	0.169	0.0097	0.96
PCC → Thal	0.098	0.0004	1.0
Temp → Temp	0.18	0.0023	1.0

References:

1. Friston KJ, Kahan J, Biswal B, & Razi A (2014) A DCM for resting state fMRI. *Neuroimage* 94:396-407.
2. Beggs JM & Plenz D (2003) Neuronal avalanches in neocortical circuits. *J Neurosci* 23(35):11167-11177.
3. Shin CW & Kim S (2006) Self-organized criticality and scale-free properties in emergent functional neural networks. *Phys Rev E Stat Nonlin Soft Matter Phys* 74(4 Pt 2):045101.
4. Stam CJ & de Bruin EA (2004) Scale-free dynamics of global functional connectivity in the human brain. *Hum Brain Mapp* 22(2):97-109.
5. Razi A & Friston K (2016) The Connected Brain: Causality, models, and intrinsic dynamics. *IEEE Signal Processing Magazine* 33(3):14-35.
6. Friston KJ, Harrison L, & Penny W (2003) Dynamic causal modelling. *Neuroimage* 19(4):1273-1302.
7. Friston K, Mattout J, Trujillo-Barreto N, Ashburner J, & Penny W (2007) Variational free energy and the Laplace approximation. *Neuroimage* 34(1):220-234.
8. Friston KJ, *et al.* (2016) Bayesian model reduction and empirical Bayes for group (DCM) studies. *Neuroimage* 128:413-431.
